# Potential Implications of Quercetin and its Derivatives in Cardioprotection

**DOI:** 10.3390/ijms21051585

**Published:** 2020-02-26

**Authors:** Kristina Ferenczyova, Barbora Kalocayova, Monika Bartekova

**Affiliations:** 1 Institute for Heart Research, Centre of Experimental Medicine, Slovak Academy of Sciences, 84104 Bratislava, Slovakia; kristina.ferenczyova@savba.sk (K.F.); barbora.kalocayova@savba.sk (B.K.); 2 Institute of Physiology, Comenius University in Bratislava, 81372 Bratislava, Slovakia

**Keywords:** quercetin (QCT), QCT derivatives, cardioprotection

## Abstract

Quercetin (QCT) is a natural polyphenolic compound enriched in human food, mainly in vegetables, fruits and berries. QCT and its main derivatives, such as rhamnetin, rutin, hyperoside, etc., have been documented to possess many beneficial effects in the human body including their positive effects in the cardiovascular system. However, clinical implications of QCT and its derivatives are still rare. In the current paper we provide a complex picture of the most recent knowledge on the effects of QCT and its derivatives in different types of cardiac injury, mainly in ischemia-reperfusion (I/R) injury of the heart, but also in other pathologies such as anthracycline-induced cardiotoxicity or oxidative stress-induced cardiac injury, documented in in vitro and ex vivo, as well as in in vivo experimental models of cardiac injury. Moreover, we focus on cardiac effects of QCT in presence of metabolic comorbidities in addition to cardiovascular disease (CVD). Finally, we provide a short summary of clinical studies focused on cardiac effects of QCT. In general, it seems that QCT and its metabolites exert strong cardioprotective effects in a wide range of experimental models of cardiac injury, likely via their antioxidant, anti-inflammatory and molecular pathways-modulating properties; however, ageing and presence of lifestyle-related comorbidities may confound their beneficial effects in heart disease. On the other hand, due to very limited number of clinical trials focused on cardiac effects of QCT and its derivatives, clinical data are inconclusive. Thus, additional well-designed human studies including a high enough number of patients testing different concentrations of QCT are needed to reveal real therapeutic potential of QCT in CVD. Finally, several negative or controversial effects of QCT in the heart have been reported, and this should be also taken into consideration in QCT-based approaches aimed to treat CVD in humans.

## 1. Introduction

During the last decades, constantly growing interest of the effects of flavonoids and other polyphenols on human health has been noticed. Flavonoids are a group of polyphenolic compounds present in the diet representing a promising therapeutic and/or preventive agents for a variety of diseases including cardiovascular disease, diabetes mellitus, hypertension and cancer [1,2,3,4].

Quercetin (QCT) is a common flavonoid highly enriched in frequently consumed fruits, vegetables and berries. Major natural sources of QCT and its derivatives are onions, peppers, plums, mangos and various types of berries. Extensive research is focused on exploring the beneficial effects of QCT for human health at all body systems including cardiovascular, nervous, gastrointestinal and others, as well as on uncovering molecular mechanisms involved in QCT action in the body. By its antioxidant, anti-inflammatory, anti-thrombotic, anti-apoptotic and other effects [5], QCT possesses a wide range of multiple activities influencing many different signaling pathways. Thus, QCT affects a number of physiological processes, and is believed to be beneficial in various human diseases including cancer, obesity and diabetes, gastrointestinal and renal diseases [6,7,8].

In cardiovascular system, QCT and certain QCT-containing food have been shown to exert strong anti-hypertensive effects in both experimental animals and humans through numerous mechanisms such as attenuation of oxidative stress, affecting intracellular protein kinase cascades, as well as via remodeling of extracellular matrix in the vasculature [9,10,11,12].

In addition to its vascular effects, QCT has been shown to exert robust heart-protective effects in different kinds of cardiac injury, including ischemia-reperfusion (I/R) injury, doxorubicin-induced cardiotoxicity, diabetic cardiomyopathy and others [13,14,15,16,17]. Cardioprotective effects of QCT are associated with affecting many different signaling pathways and proteins, including inhibition of apoptosis and decreasing oxidative stress, as well as affecting inflammatory proteins in the heart [14,15,16,18].

In line with increasing evidence of beneficial effects of QCT in different types of heart disease, the aim of the present review is to summarize current knowledge on potential cardioprotective effects of QCT and its derivatives in different types of cardiac injury. The paper focus mainly on the recent experimental studies exploring effects of this flavonoid in in vitro as well as in vivo models of cardiac injury, and provide detailed information about proposed mechanisms involved in cardiac effects of QCT and its derivatives. Finally, potential difficulties of QCT use in humans are outlined in the paper, including potential confounding factors that may affect QCT efficiency in preventing cardiac injury.

## 2. QCT and its Derivatives: Structure, Sources, Metabolism, Bioavailability

**Figure 1:**
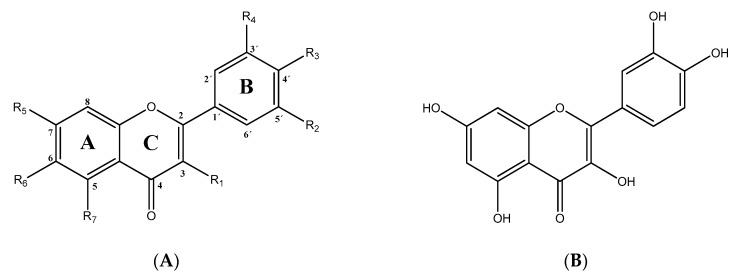
Chemical structures of: (**A**) flavone backbone with potential substituent sites; (**B**) QCT.

QCT (2-(3,4-dihydroxyphenyl)-3,5,7-trihydroxy-chromen-4-one) (IUPAC name) is one of the major representatives of the flavonol family, a subgroup of flavonoids, compounds characterized by 3-hydroxyflavone backbone (Figure 1A). QCT is considered a strong antioxidant possessing the ability to scavenge free radicals and to bind transition metal ions [19]. The catechol and the OH groups at position C3 give QCT the optimal configuration for free radical scavenging. All these properties are primarily attributed to the presence of two antioxidant pharmacophores within the molecule. Despite its attractive molecule shape and preferences, some limitations complicate the use of QCT as a drug. In fact, bioavailability of QCT aglycone defined as the portion of an initially administered dose that reaches the systemic circulation unchanged after a single oral dose was estimated at 4%, which is very low, mostly due to its fast and extensive metabolism. The factors that most influence and usually improve bioavailability of quercetin are the properties of attached sugar moieties and its solubility in water or fats [20]. In addition to its low bioavailability, QCT has low water solubility (0.01 mg/mL (25 C)) [21], high chemical instability and short biological half-life (the average terminal half-life of QCT is 3.5 h [22]), which could reduce its efficacy when it is used in the food and pharmaceuticals [23].

### 2.1. Chemistry of QCT and its Derivatives

QCT molecule is formed of a 15-carbon skeleton consisting of two phenyl rings (A and B) typical for flavonols, attached by an oxygen-containing heterocyclic ring (C). Common feature of flavonols is the hydroxyl group on C-3 carbon [24]. QCT molecule itself occurs as an aglycone with five hydroxyl groups on the flavone backbone (Figure 1B). Hydroxyl groups determine reactivity and biological activity of QCT, and limit its ability to create derivatives. Despite the presence of hydroxyl groups, QCT molecule has lipophilic character, while its derivatives may become more hydrophilic [25]. 

In contrast to other food supplements, QCT is mostly bound to a saccharide in nature. This conjugate is known as a QCT glycoside. While glycosylation of at least one hydroxyl group increases hydrophilicity of QCT derivatives, binding of alkoxyl groups or alkylation maintains the lipophilic character of the molecule [26,27]. In plants, changing the profile of a QCT molecule from lipophilic to hydrophilic is aimed to increase its solubility in the cytosol of cells. Consequently, soluble molecules are more easily transported to different parts of plant, thus increasing the possibility of their storage in vacuoles [28,29].

There is a clear correlation between the structure of a QCT molecule and its antioxidant activity. Higher occupancy of hydroxyl groups by saccharides leads to lower antioxidant activity of QCT derivative. Therefore, QCT is the most effective antioxidant among all QCT derivatives since no hydroxyl group are occupied in a QCT molecule [19,30]. QCT, as well as its derivatives, are usually found in the form of yellow-colored powder or small crystals, and cannot be synthetized in the human body [31]. Molecules derived from QCT are classified as: (1) O-glycosides, (2) C-glycosides, (3) ethers, (4) derivatives containing alkyl substituents (prenyls) (Table 1).

#### 2.1.1. QCT-O-Glycosides

In nature, QCT is widely distributed in O-glycoside form with one or more hydroxyl groups replaced by different saccharides. QCT-3-O-glycosides are largely present in fruits, vegetables and the anatomical parts of plants. In these derivatives, the hydroxyl group on C-3 carbon is glycosylated by monosaccharides such as glucose, galactose, xylose or rhamnose [29]. Significant quantities of hyperoside (QCT 3-O-galactoside) were found in mango [32] and small fruits, especially cranberries, blueberries and chokeberries [33]. Quercitrin (QCT-3-O-rhamnoside) was detected in mango [32] and spinach [34]; isoquercitrin (QCT-3-O-glucoside) was found in beans [35], plums [36], onions [37] and mango [32]. QCT derivatives with more complex saccharides bound to C-3 hydroxyl group can also be found in plant foods, namely rutin with terminal sugar rutinose (disaccharide), which is found in abundance in plums [36], cherries [38], tomatoes [39] and buckwheat [40], and QCT-3-O-sophoroside (disaccharide sugar moiety), which is found in broccoli [41]. 

Hydroxyl group on C-7 carbon of QCT molecule can be also O-glycosylated, as in the case of QCT 7-O-glucosid found in beans [35]. 7-O-glycosylation is often accompanied by methylationon C-3 carbon, for instance in QCT-3-O-rhamnoside-7-O-glucoside found in pepper fruit [42]. In nature, however, a number of glycosides derived from QCT can be found, since sugar moieties can form additional bonds and bind substituents such as acyls (links with aliphatic acids, e.g., malonic, acetic, aromatic acids including caffeic or benzoic acids) or sulfates (SO_4_^2−^) [25]. An example of acyl derivative is QCT-3-O-(2′-acetylgalactoside) [43]. QCT-3-O-glucoside-5′-sulfate identified in the cornflower [44] is one of the few representatives of QCT sulfates that are only rarely found in nature.

#### 2.1.2. QCT-C-Glycosides

Another group of QCT derivatives are C-glycosides, which are very rare in nature. Glycosylation usually occurs on C-6 carbon. An example of such a derivative is QCT-6-C-glucoside, which was originally found in plant *Ageratina calophylla* [25].

#### 2.1.3. QCT Ethers

In the third group of QCT derivatives, a bond is formed between the alcohol molecule and any hydroxyl group of the QCT molecule, most often methanol. Representatives of this group can be found in food, such as isorhamnetin (3-O-methylQCT) enriched in onions and honey [46,47], and rhamnetin (7-O-methyl QCT) enriched in berries *Rhamnus petiolaris* [45]. 

#### 2.1.4. Alkyl-Containing QCT Derivatives (Prenyls)

The last group of QCT derivatives is only very rarely described in the literature, and its representatives have not been tested for their cardiovascular effects thus far. Thus, this group has minor importance in our overview. An example of a QCT derivative of this group is 8-prenyl-QCT, present in *Desmodium caudatum* [48].

### 2.2. Metabolization of QCT in the Body

Depending on the substituent on the QCT backbone, absorption of QCT derivatives occurs in different parts of the gastrointestinal tract. It has been shown that QCT in the form of aglycone, in contrast to its glycoside forms absorbed primarily in the intestine, is absorbed already in the stomach. However, the mechanism of absorption in the stomach still remains unknown [49]. Investigations performed in human-derived Caco-2 cells, a model of epithelial cells of intestinal absorption, revealed higher permeability of QCT aglycone as compared to QCT glycosides via cell monolayer by simple passive diffusion in the small intestine [50]. This observation correlates with the fact that QCT is more lipophilic than its hydrophilic derivatives [51]. Hydrolysis of the glycosidic bond of QCT monosaccharide derivatives (such as isoquercitrin) occurs in the lumen of the small intestine by the activity of lactase-phlorizin hydrolase (LPH), a β-glucosidase located at the apical membrane of enterocytes. This results in formation of QCT aglycone which enters the enterocyte by simple diffusion [52].

Studies have shown that glucose-linked QCT derivatives are transported by another mode of transport from the small intestinal lumen to the enterocyte cytosol, by a sodium-dependent glucose cotransporter-1 (SGLT-1) [53]. When glucose-linked QCT derivatives enter into the enterocyte, their molecules are degraded to QCT aglycone and glucose by cytolosic β-glucosidase. QCT oligosaccharides and polysaccharides as well as monosaccharide derivatives, which have not been absorbed or processed yet are deglycosylated in more distal intestinal parts—the large intestine (colon) by microbiota-derived β-glucosidase back to QCT aglycone [54], which is subsequently absorbed or degraded to phenolic acids [55]. Enterobacteria responsible for QCT metabolization in the colon belong to different strains, for instance, *Clostridium orbiscindens* [56]. As followed, hydrolysis of QCT glycosides to QCT aglycone is essential for their efficient absorption, either in enterocytes or by enterobacteria. QCT aglycone from both small and large intestine in enterocytes/colonocytes. Moreover, QCT aglycone in enterocytes presents a subject to enzymes of phase II metabolism catalyzing conjugation reactions (glucuronidation and/or sulfate conjugation) by UGT (UDP-glucuronosyltransferase) [57], SULT (sulfotransferase) [58] and modification reactions (O-methylation) by COMT (catechol-O-methyltransferase) [59], mostly before entering the portal vein via ATP-binding cassette (ABC) transporters [60]. This was investigated by studies where the presence of residues of unmetabolized QCT algycone was confirmed, but mostly QCT methylated and/or unmethylated QCT metabolites (glucuronides and sulphates) were found in human plasma [61], lymph [62] and in portal vein [63]. The most commonly present methylated phase II QCT metabolites include isorhamnetin and tamarixetin [64] or unmethylated QCT 3-O-β-D-glucuronide [65]. QCT metabolites, which enter the liver by passive diffusion or by organic anion transporters (OATs), are extensively exposed to further reactions catalyzed by phase II metabolism enzymes. Subsequently, they are excreted into the bloodstream for further action in the body or directed to the bile [5,63].

**Figure 2:**
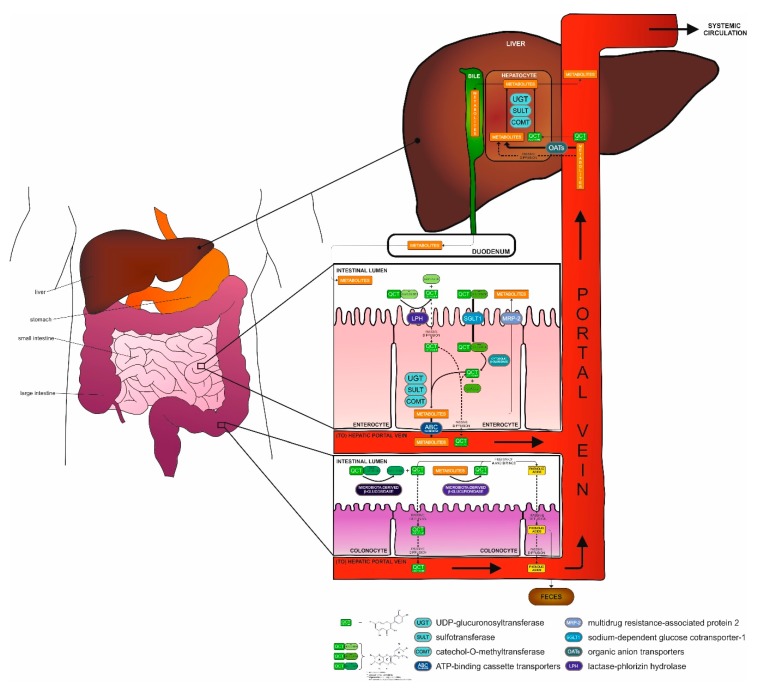
Overview of QCT metabolization in the body. QCT and its monosaccharide* derivatives (including QCT glucoside**) are metabolized in small intestine. After a chain of reactions catalyzed by enzymes UGT, SULT or COMT causing glucoronidation, sulfation or methylation, respectively, QCT metabolites are either transported by ABC transporters to the portal vein and then to liver or re-uptake and transport back to the intestinal lumen by MRP-2, continuing to the large intestine. QCT aglycone as a possible product of QCT glycosides and QCT glucosides is transferred by passive diffusion through enterocytes to hepatic portal vein and consequently to the liver. In the large intestine, mainly QCT oligosaccharides and polysaccharides (QCT glycosides***) are enzymatically deglycosylated to QCT aglycone, which is transported from intestinal lumen to portal vein by passive diffusion through colonocytes. Degradation of QCT metabolites, which were transported from the small intestine to the large intestine, occurs in the large intestinal lumen, where they are degraded to phenolic acids. In the liver, further metabolization of thus far created QCT metabolites or QCT aglycone occurs by their conjugation (by UGT or SULT) or modification (by COMT). Finally, QCT metabolites are transported from liver to either systemic circulation or back to duodenum (small intestine) via bile, possibly heading to large intestinal final degradation. For more details, see Chapter 2.2.

The elimination phase III of metabolism begins in liver by excretion of QCT metabolites to bile continuing to duodenum and also in the small intestine itself, where the metabolites are transported back to the intestinal lumen by MRP-2 protein (multidrug resistance-associated protein 2) [66]. Thus, in large intestine, in addition to deglycosylation by microbiota-derived β-glucosidase [67], there occurs a final degradation of unabsorbed QCT derivatives as well as QCT metabolites, which were transported back to intestine via bile. Degradation involves deconjugation and deglycosylation of QCT metabolites to QCT aglycone with the aim of fission of the A and B-rings of the QCT backbone, leading to the formation of low molecular weight phenolic acids. This is also confirmed by the presence of microbiota-derived β-glucuronidase in large intestine microflora, which, after deglucuronidation, provides QCT aglycone for further degradation [55]. The most common degradation products, such as 3,4-hydroxyphenylacetic acid, hippuric acid but also QCT aglycone itself, are either re-absorbed into the bloodstream circulation or excreted by feces from the body [68]. An overall picture of QCT metabolization is outlined in Figure 2.

## 3. QCT and its Derivatives as Cardioprotective Agents

One of the major therapeutic goals of modern cardiology is to design strategies aimed at saving myocardium from the negative effects of ischemia-reperfusion (I/R) injury associated with such pathological states as ischemic heart disease and acute myocardial infarction, the major types of cardiovascular disease (CVD) and top causes of death worldwide. QCT, as well as several other natural polyphenols, has been documented to exert beneficial effects in CVD, including cardiac I/R injury. The cardioprotective activity of QCT and its derivatives in patients suffering from ischemic heart disease (IHD) is enforced, and was repeatedly confirmed in experimental studies performed in both cellular and animal models of cardiac I/R injury. A potential mechanism of QCT action in the heart has also been extensively studied. In addition, there is an urgent need to develop therapeutic strategies against non-ischemic cardiac pathologies, such as various cardiomyopathies of different origin. In line with this need, cardioprotective potential of QCT has also been explored in experimental models of non-ischemic cardiac diseases. In this section cardioprotective effects of QCT documented in various experimental models of cardiac damage are reviewed.

### 3.1. In Vitro and Ex Vivo Cardioprotection Afforded by QCT and QCT-Rich Plants

Cardioprotective effects of QCT have been documented in numerous models of in vitro cardiomyocyte injury. In the model of 4-hydroxynonenal-induced toxicity in H9c2 cardiac-derived cell line (4-hydroxy-2-nonenal is a secondary product of lipoperoxidation, and can form protein adducts and modifies cell signaling), QCT pretreatment (0.1–10 µM for 24 h) decreased ROS production, p-SAPK/JNK levels, p-Hsp27 levels, caspase-3 expression and improved cell viability, thus ameliorating in vitro oxidative damage to rat cardiomyocytes [69]. A study of Chen et al. [70] demonstrated that 4-h pretreatment with QCT in different concentrations (50–200 μM) reduced cardiotoxicity in cancer chemotherapy-induced cell damage in H9c2 cells during 24 h exposure to 0.45 μM doxorubicin. Moreover, application of its methanol extract alligator weed (*Alternanthera philoxeroides*), a plant rich in QCT (10–160 mg/mL, for 24) prevented cardiomyocyte apoptosis induced by doxorubicin in H9c2 cells [71]. Naturally occurring QCT is also present in *Syzygium cumini* seeds. One-day lasting incubation of H9c2 cells with extract from this plant (1–500 µg) protected cells against tertiary butyl hydrogen peroxide (TBHP)-induced oxidative stress [72]. QCT pretreatment (10–16 µM) proved its cardioprotective effects in H9c2 cells subjected to hypoxia/reoxygenation (H/R) (4 h/6 h) by inhibition of JNK (c-Jun N-terminal Kinase) and p38 mitogen-activated protein kinase signaling pathways and modulated the expression of Bcl-2 (B-cell lymphoma 2) and Bax (Bcl-2-associated X) proteins [73]. Pretreatment (24, 48 and 72 h) of neonatal rat primary cardiomyocytes with QCT (10–80 µM) before anoxia/reoxygenation (4 h/2 h) improved cell survival rate, decreased ROS generation, avoided collapse of the mitochondria membrane potential, inhibited the opening of mitochondrial permeability transition pores (mPTP) and alleviated subsequent apoptosis in injury. The authors of this study also hypothesized that cardioprotective effects of QCT may be mediated via enhancing protein expression of PKCε and ameliorating the activity of downstream mediators of its pathway [74]. Furthermore, addition of QCT (20 μM) to culture medium increased the cell viability of H9c2 cells with LPS induced inflammation [75].

It is known that QCT, like other antioxidants, is very rapidly metabolized in the organism, thus, the application form of QCT might play an important role in its effects. It was documented that 24-h pretreatment with encapsulated QCT into poly(lactic-co-glycolic) acid (PLGA) nanoparticles had a cardioprotective effect in H9c2 cells exposed to H/R injury (3 h/1.5 h). Encapsulated PLGA-QCT (5 μM) protected cells more effectively than free QCT (5 μM), likely due to lower oxidized thiols, maintaining the mitochondrial oxygen consumption rate and membrane potential, which sustain superior ATP production that leads to the preservation of mitochondrial function and ATP synthesis [76]. Combined treatment with QCT and resveratrol encapsulated in Pluronic^®^ F-127 micelles (mRQ) (RES:QCT in 1:1 molar ratio, capable of retaining 1.1 mg/mL of resveratrol and 1.42 mg/mL of QCT, respectively) showed new possible strategy to eliminate acute doxorubicin-induced cardiotoxicity in vitro in H9c2 cells via scavenging of ROS and decreasing caspase 3/7 activity [77].

In addition to cell culture models in cardiac-derived cells, in vitro effects of QCT have been examined in isolated heart models of I/R injury (ex vivo models). We have documented that acute administration of QCT (15 mmol/L infusion for 15 min before the onset of ischemia or during whole reperfusion, respectively) improved recovery of cardiac function after global I/R (25 min/2 h) in Langendorff-perfused rat hearts and reduced infarct size in these hearts [13]. Administration of QCT (1 mg/kg) into Krebs-Henseleit buffer during reperfusion period improved function of Langendorff-perfused rat hearts after I/R injury (30 min/30 min) through inhibition of the HMGB1 (High mobility group box-1) pathway [78].

### 3.2. In Vitro Cardioprotection Afforded by QCT Derivatives

In addition to QCT alone, QCT derivatives were documented to exert cardioprotection in different experimental settings simulating cardiac injury. Pretreatment of neonatal rat cardiomyocytes (NRCMs) with isorhamnetin (3′-O-methyl-QCT; 10–40 mM) 24 h before anoxia/reoxygenation (3 h/2 h) increased cell viability and expression of SIRT1, reduced the generation of ROS, inhibited opening of mPTPs, reduced the loss of Δψm and decreased the activation of caspase-3 and release of cytochrome c thus reducing apoptosis, and finally, reduced the the release of lactate dehydrogenase and creatine phosphokinase from cardiomyocytes [79]. 12-h pretreatment with dihydro-QCT (2.5–80 μM) protected H9c2cells against H/R injury (H-6 h/R-16 h) by inhibiting oxidative stress- and endoplasmic reticulum stress-induced apoptosis via activation of the PI3K/Akt pathway [80]. Another QCT derivate, ZYZ-772 (QCT-3-O-(6″-O-α-l-rhamnopyransoyl) -β-d-glucopyranoside-7-O-β-d-glucopyranoside; 1–50 µM for 2 h) protected H9c2 cells against CoCl_2_-induced H/R (12 h/4 h) injury. It is suggested that ZYZ-772 protected cells by suppression of Nox4/MAPK/P53 axis in conditions of CoCl_2_-induced hypoxia injury [81]. Hypoxia-induced apoptosis of NRCMs was attenuated by pretreatment with hyperoside (QCT-3-O-galactoside; 0.5–50 μM for 12, 24, 36 h) in an in vitro model of cardiac H/R (8 h/2 h) injury, likely through suppression of the Bnip3 expression [82]. Perfusion of isolated hearts with dihydro-QCT (5–20 μM) added into the Krebs–Henseleit solution for 20 min prior to I/R (45 min/50 min) protected hearts by inhibiting oxidative stress- and endoplasmic reticulum stress-induced apoptosis via the PI3K/Akt pathway [80]. It was documented that 24-h lasting supplementation with isoquercetin (isoquercitrin, isoQCT; 20–80 μg/mL) increased cell viability of H9c2 cells after I/R (6 h/12 h) injury by protection of mitochondrial function and prevention of cytochrome c release [83]. In the study of Daubney et al. [84], the effects of 24 h pretreatment of H9c2 cells with QCT and two of its major metabolites QCT-3-glucuronide and 3′-O-methyl-QCT prior to 2-h exposure to 600 µM H_2_O_2_ were monitored. As a result, QCT triggered cardioprotection against oxidative stress-induced cell death via attenuation of H_2_O_2_-induced activation of ERK1/2, PKB, p38 MAPK and JNK. On the other hand, inhibitors of these kinases did not modulate QCT-induced protection against H_2_O_2_-induced cell death. Interestingly, cardioprotection was observed with QCT and 3′-O-methyl-QCT, but not with QCT-3-glucuronide.

### 3.3. In Vivo Cardioprotection Afforded by QCT and QCT-Rich Plants

Cardioprotective potential of QCT has been widely documented in different in vivo animal models of cardiac injury. It was suggested that phytochemical QCT may play a key role in cardioprotection and help in remodeling of the heart during isoproterenol-induced cardiac ischemia and fibrosis [85]. Two weeks of QCT pretreatment (50 mg/kg) of rats with isoproterenol-induced myocardial infarction induced cardioprotective effects manifested by significantly attenuated oxidative stress, inflammation, as well as protected heart architecture. These effects of QCT were associated with downregulation of the expression of calpain [86]. QCT was shown beneficial also in Duchenne muscular dystrophy, a juvenile musculoskeletal genetic disease associated with progressive cardiac pathology. In an animal model of muscular dystrophy, long-term dietary QCT enrichment (0.2%) improved cardiac function in aged Mdx/Utrn^+/−^ mice (lack of dystrophin and heterozygous knockout for utrophin; aged Mdx/Utrn^+/−^ mice exhibit accelerated declines in cardiac health and dystrophic pathology) and increased mitochondrial protein content and dystrophin glycoprotein complex formation [87]. Treatment of Lewis rats with QCT (10 mg/kg) protected against progression of experimental autoimmune myocarditis by suppression of oxidative and endoplasmic reticulum stress via endothelin-1/MAPK signaling. In the study, myocardial dimensions and cardiac function were preserved significantly in the QCT-treated rats in comparison with the rats treated with vehicle [88]. QCT pretreatment (4 weeks in a dose 25 mg/kg, once-daily gavage) also significantly reduced cardiac mitochondrial H_2_O_2_ production, total content of Ca^2+^ in cardiac tissue and collagen volume fraction in a model of cardiac injury induced by chronic aldosterone/salt treatment in male Sprague-Dawley rats, which is typically accompanied with adverse structural remodeling of myocardium [89].

In addition to effects of QCT supplementation in pure form, 60-days treatment with *Phyllanthus amarus* (plant reach in QCT) showed protection of the heart from high fructose-diet induced damage. The *Phyllanthus amarus* treatment protected male Wistar rats from high fructose-diet-induced increase in stress markers and a decrease in non-enzymatic and enzymatic antioxidants in the heart and aorta [90]. An interesting form of QCT administration was used in study Cote et al. [77], where a combination of QCT with Resveratrol in Pluronic^®^ F-127 micelles (mRQ) (RES:QCTin 1:1 molar ratio, capable of retaining 1.1 mg/mL of Resveratrol and 1.42 mg/mL of QCT, respectively) was prepared for application. In vivo treatment of mice with mRQ conferred full cardioprotection in doxorubicin-induced cardiotoxicity [77]. To elucidate molecular signaling pathways involved in QCT-induced cardioprotection male Wistar albino rats with sodium nitrite-induced hypoxia were used. Pretreatment of hypoxic rats with QCT (200 mg/kg, i.p.) was accompanied with down-regulation of mRNA expression of nuclear factor kappa-B (NF-κB), Bax, and flt-1 and suppressed DNA damage. Thus, QCT effectively declined the cardiotoxic effects of sodium nitrite and ameliorated cardiac injury in these rats [91].

It is well known that homeostasis of the endoplasmic reticulum and its correct function is disrupted in various types of cardiac disease. Interestingly, QCT is a substance capable to activate IRE1 (Inositol-requiring transmembrane kinase/endoribonuclease 1), an important transmembrane protein of endoplasmic reticulum [92,93], thus, potentially influencing the function of endoplasmic reticulum under stress conditions. It was documented that the p21-activated kinase 2 (Pak2)-cardiac deleted mice (Pak2-CKO) exhibited impaired function of endoplasmic reticulum, dysfunction of the heart and serious cell death due to tunicamycin treatment-induced stress or pressure overload. Administration of QCT (10 mg/kg/day, daily gavage) for 2 weeks alleviated malfunction of endoplasmic reticulum in Pak2-CKO hearts the second day after transverse aortic constriction [94].

In addition to the above mentioned types of cardiac injury, cardioprotective effects of QCT have been widely documented in several in vivo models of myocardial ischemic injury. In an in vivo rat model of cardiac I/R injury (30 min/4 h), orally given QCT (250 mg/kg for 10 days) decreased oxidative stress, repressed inflammatory cascade, inhibited apoptosis and activated the PI3K/Akt pathway (involved in the anti-apoptotic effect) in the heart tissue [16]. With the same dose, QCT administration for 10 days suppressed the NF-κB pathway via up-regulating PPARγ expression in mice exposed to simulated I/R (30 min/24 h) [95]. Treatment of rats with QCT (1 mg/kg/day) induced a significant reduction of infarct size and improved hemodynamic abnormalities in hearts subjected to 30 min ischemia by left coronary artery occlusion followed by 12 h reperfusion. QCT treatment also decreased expression of both tumor necrosis factor-alpha (TNF-α) and interleukin-10 (IL-10) and lowered serum levels of inflammatory cytokines, suggesting anti-inflammatory effects of QCT in preventing cardiac I/R injury [96]. One-week treatment of female Sprague Dawley rats with QCT (25–100 mg/kg, gavage, daily) protected rats against coronary artery ligature-induced I/R (30 min/2 h) injury via an increased SIRT1/PGC-1a pathway and Bcl-2/Bax ratio [97].

In our studies, we have also documented several anti-ischemic effects of chronic in vivo QCT administration. 4-weeks lasting in vivo oral treatment with QCT (20 mg/kg/day) improved post-ischemic (25 min/40 min) recovery of heart function of isolated rat hearts from juvenile but not from adult Wistar rats [13]. We have also shown that prolonged in vivo QCT treatment (20 mg/kg/day for 6 weeks) significantly improved post-ischemic recovery of heart function of isolated hearts from both healthy and doxorubicin-treated rats [14]. Importantly, QCT not only protected hearts against I/R injury, but also reversed doxorubicin-induced detrimental changes in the heart tissue including ultrastructural changes, matrix metalloproteinase-2 activation and apoptosis induction [14].

### 3.4. In Vivo Cardioprotection Afforded by QCT Derivatives

Regarding in vivo effects of QCT derivatives in cardiac injury, it was documented that two weeks lasting treatment with isoQCT (80 mg/kg/day by gavage) protected male Sprague-Dawley rat hearts against acute myocardial infarction *in vivo*. IsoQCT protected myocardium through anti-inflammatory and anti-apoptotic effects, and via regulation of the TLR4-NF-κB signaling pathway [98]. Pretreatment of male Wistar rats with another QCT derivative troxerutin ((3′,4′,7-Tris[O-(2-hydroxyethyl)]rutin; 150 mg/kg for one month) protected myocardium against I/R injury (30 min/45 min) maintained by ligation of the left anterior descending artery. Rats treated with troxerutin exhibited significantly reduced myocardial infarct size, improved cardiac function likely via the modulated PI3K/Akt pathway [99]. Concordantly, treatment of male Wistar rats with troxerutin (150 mg/kg daily for one month) protected isolated hearts against I/R (30 min/45 min) injury via the inhibition of myocardial inflammatory cytokines TNF-α and IL-1β and inhibited activation of leukocyte-endothelial cell interaction molecule (ICAM-1) after I/R insult [100]. Plants such as stonebreaker (*Phyllanthus amarus*) and bitter gourd (*Momordica charantia*) represent good sources of antioxidants and QCT, as well as its derivatives quercitrin, isoquercitrin and rutin. Supplementation of extracts from these plants (each 200 and 400 mg/kg for 2 weeks by gavage) protected male Wistar albino rats against doxorubicin-induced cardiotoxicity by reversing redox imbalance and by modulating biomolecules associated with worsened cardiac function altered by doxorubicin, such as angiotensin-converting enzyme, arginase, acetylcholinesterase and adenosine deaminase [101].

Proposed cardioprotective effects of QCT and its derivatives documented in experimental studies are summarized in the Table 2.

## 4. Role of Comorbidities in Cardioprotection by QCT and its Derivatives

In the previous parts of this review, QCT was tested for its cardioprotective effects more or less exclusively in healthy animals or standard cell cultures. However, the presence of different comorbidities in individuals suffering from heart disease might influence the efficacy or even reverse effects of treatments aimed to prevent cardiac injury, including cardioprotective effects of QCT. Moreover, treatment may influence comorbidity itself and thus evoke different mechanisms and effects than those afforded in the absence of comorbidities. In line with this view, we provide a short overview of cardiac effects of QCT in presence of comorbidities documented thus far. In addition, potential influence of QCT on the progression of comorbidity itself, e.g., diabetes, will be discussed as well. One of the major complications of *Diabetes mellitus* is diabetic cardiomyopathy [105]. Bioactive compounds such as QCT have been shown to exert beneficial effects in ameliorating the pathogenesis of diabetic cardiomyopathy. The 28 days lasting administration of QCT (10–50 mg/kg) to Sprague Dawley male rats with streptozotocin (STZ)-induced diabetes caused significant decrease of cardiac injury markers levels, particularly troponin-C, creatine kinase-isoenzyme (CK-MB) and lactate dehydrogenase (LDH). In addition, ameliorated histopathological changes, oxidative stress, inflammation and apoptosis levels were observed [103]. In the study of Soman et al. [106], it was found that pure QCT (50 mg/kg) as well as extract from *Psidium guajava* (a plant highly enriched with QCT) showed beneficial effects on the diabetic heart. After the induction of diabetes by STZ (55 mg/kg) in female Sprague Dawley rats, QCT or *Psidium guajava* extract, respectively, was administered for 60 days. Both treatments were accompanied with decreased levels of AGEs (advanced glycation end products) in the diabetic heart suggesting beneficial cardiac effects of QCT in diabetic subjects [106]. Moreover, cardioprotective effects of QCT and rutin (QCT derivative) were documented in I/R-induced myocardial infarction in both normal and diabetic rats. Albino Wistar rats with STZ-induced diabetes (45 mg/kg) were treated with QCT or rutin (5–10 mg/kg, i.p.) 10 min before the onset of reperfusion. After I/R (30 min/4 h) induced by coronary artery occlusion it was documented that the heart of rats treated with QCT or rutin, respectively, exhibited significantly lower infarct sizes in both normal and diabetic animals in a similar approach [102]. Finally, hearts from STZ-diabetic Male Wistar rats treated with troxerutin (150 mg/kg, daily gavage) for 4 weeks were exposed to I/R injury on Langendorff aparatus (30 min/60 min). Troxerutin pretreatment improved myocardial function after I/R injury in both healthy and diabetic rat hearts likely through anti-arrhythmic and anti-inflammatory effects [104].

Hypercholesterolemia is another major risk factor for the development of myocardial damage. It is suggested that QCT could be effective modulator of plasma cholesterol and may have protective effect in cardiac remodeling in hypercholesterolemia. In the study of Ulasova et al. [107], 6 weeks lasting oral administration of QCT (0.1µmol/kg) markedly reduced total cholesterol and very low density lipoprotein (VLDL) levels in plasma of Apo^-/-^ hypercholesterolemic mice, a model with typically developed left ventricular hypertrophy. After QCT treatment, the hypertrophy was reduced followed by deceased left ventricle posterior wall thickness and left ventricle mass [107]. *Crataegus pinnatifida* is fruit rich in polyphenols, among others also rutin and isoquercitrin. It was documented that high-fat diet fed atherosclerotic rats supplemented with extract from the *Crataegus pinnatifida* (72 and 288 mg/kg) via the intragastric route for 4 weeks had lower plasma levels of lipids (total cholesterol, total triglycerides, LDL-cholesterol, HDL-cholesterol), decreased inflammatory response and inhibited pathological changes in the arteries of atherosclerotic rats suggesting potential of the *Crataegus pinnatifida* to reduce the development of cardiovascular diseases [108]. On the other hand, our recent study [109] documented different effects of QCT on vasculature and the heart in *Diabetes mellitus* type 2. In the study, 6-month and 1-year-old male Zucker diabetic fatty rats (ZDF) were daily treated with QCT (20 mg/kg) for 6 weeks. QCT exerted age-dependent beneficial effects on vascular function and blood pressure but was inefficient in preventing myocardial I/R (30 min/2 h) injury in ZDF rats [109].

## 5. Cardiovascular Effects of QCT in Human Studies and Clinical Trials

Up to now (February 19, 2020), 70 QCT clinical trials have been registered at ClinicalTrials.gov, a database of privately and publicly funded clinical studies conducted worldwide (available online: https://clinicaltrials.gov/ct2/results?term=quercetin). However, only few of them were aimed to reveal the cardiovascular effects of QCT; moreover, not all of them examined effects of QCT alone; some effects of different QCT-containing mixtures have been used as well.

Despite reports that increased risk of coronary heart disease (CHD) in some populations is associated with very low dietary supply of flavonoids (among others also QCT) [110], only a very limited number of human studies were focused directly on cardiac effects of QCT. Among them, it was documented that QCT possesses anti-ischemic and anti-arrhythmic effects, and exerts a regulating influence on vegetative homeostasis in patients with a chronic form of IHD with metabolic syndrome [111]. In patients with stable coronary heart disease (CHD), QCT (120 mg/day for 2 months, p.o.) significantly improved the left ventricular (LV) systolic function in terms of ejection fraction and improved LV diastolic function in terms of the ratio of the phases of the transmitral flow. Moreover, 24-h Holter electrocardiographic (ECG) monitoring showed decreased total time and number of episodes of ST segment depression in QCT-treated patients, altogether suggesting cardioprotective properties of QCT in CHD [112].

In addition to their cardiac effects, clinical studies focused on vascular effects of QCT and its derivatives have been performed. It was documented that 2-week QCT supplementation (500 mg/day) in 72 adult women with *Diabetes mellitus* type 2 significantly lowered systolic blood pressure; however, this had no effect on other cardiovascular risk factors and inflammatory biomarkers [113]. On the other hand, 4-week treatment with encapsulated QCT-3-glucoside (160 mg/day) in 37 healthy participants of both genders resulted in no changes in flow-mediated arterial dilation, insulin resistance or other cardiovascular risk factors [114]. A recent meta-analysis of clinical data documented that QCT supplementation (possibly limited to, or greater with dosages of >500 mg/day) significantly reduced blood pressure [115]. Finally, one-year supplementation with QCT in patients with gout, a disease associated with increased risk of cardiovascular diseases including heart failure, also suffered from essential hypertension treated with standard therapy (antihypertensive and urate-lowering regimens) improved left ventricular diastolic function, purine metabolism, renal function and normalized blood pressure [116].

## 6. Controversial Findings and Potential Cardiotoxic Effects of QCT and its Derivatives

As mentioned already, QCT exerts many biological beneficial effects including those in the cardiovascular system. However, controversial data and even cardiotoxic effects of QCT have been documented as well, and should be mentioned in this review to create an overall picture of cardiac effects of QCT.

It is known that the beneficial effects of certain cardioprotective interventions, e.g., acute as well as late phase of cardioprotection induced by ischemic preconditioning, are eliminated in hyperlipidemic hearts. In line with this, the effects of QCT and glycogen synthase kinase-3β (GSK-3β) inhibitors were tested in isolated hearts from hyperlipidemic rats (induced by 6-week lasting high-fat diet) subjected to I/R injury (30 min/2 h). GSK-3β inhibitors SB216763 (SB) and indirubin-3-monoxime (IND) were administered 24 h before, and QCT (4 mg/kg, i.p.) was given 25 h before the isolation of hearts. GSK-3β inhibitors were found to exert cardioprotective effects in I/R injury, and these effects were attenuated by QCT, manifested by increased myocardial infarct size and release of lactate dehydrogenase and creatine kinase-MB. In this study, QCT was uncommonly used as an inhibitor of HSP72 (heat shock protein 72), not as a cardioprotective compound [117]. Yao et al. [118] reported that pretreatment of rats with lipopolysaccharide (LPS) increased myocardial functional recovery in hearts exposed to I/R (30 min/3 h) induced by coronary artery occlusion, partly through inhibition of NF-κB via increase of HSP70. Administration of QCT (100 mg/kg, i.p.) two hours before I/R injury decreased cardioprotection induced by LPS. In this study, QCT was used in the role of HSP70 inhibitor and the authors hypothesized that inhibition of HSP70 could attenuate the effect of LPS pretreatment [118]. However, this study might be criticized due to the use of LPS for cardioprotection, since normally, inflammation (including LPS-induced) causes negative consequences in the heart, e.g., may induce myocarditis.

Potential cardioprotective vs. cardiotoxic effects of QCT were tested also in vitro in cultured cardiomyocytes. In the study of Daubney et al. [84] increased concentrations of QCT (1–100 µM) for 24, 48 and 72 h were applied to differentiated H9c2 cardiomyocytes. MTT viability assay and LDH release testing showed that QCT induced cardiotoxic effects, which were the most evident after 48 h treatment in 30 and 100 µM concentration of QCT. After 72 h treatment, toxic effect of QCT was visible even at 10 µM concentration of QCT. Thus, in line with one of basic principles of toxicology “The dose makes the poison” (“*Sola dosis facit venenum*” by Paracelsus, 1538), experimental studies revealing cardiac effects of QCT indicated that prolonged exposure to high doses of flavonoids may lead to detrimental effects on cardiac cells, likely due to their possible pro-oxidant effects in dependence on the actual conditions [5,119].

## 7. Conclusions and Future Perspectives

**Figure 3:**
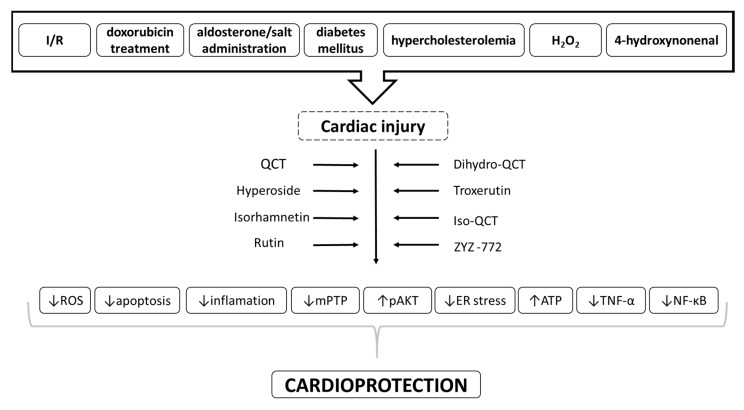
Scheme of potential cardioprotective effects of QCT and its derivatives in heart injury outlining the proposed molecular mechanisms involved in their action.

Application of QCT and its derivatives in different cell culture and animal models of cardiac injury and their potential beneficial effects in preventing cardiac dysfunction due to cardiac I/R injury as well as other cardiac pathologies has been widely documented (Figure 3). Thus, QCT and its derivatives may represent promising cardioprotective substances for prevention/treatment for wide range of cardiac disease. On the other hand, metabolic comorbidities, at least diabetes mellitus type 2, might act as confounding factors for cardioprotection by QCT. In addition, non-metabolic factors such as ageing might also act as a confounding factor for cardioprotective effects of QCT. Thus, the age of the treated subject and presence of lifestyle-related comorbidities should be taken into consideration in potential use of QCT for prevention and/or treatment of cardiovascular disease in humans.

Despite promising experimental results pointing to potential beneficial cardiovascular effects of QCT, the results from human studies are still inconclusive due to very small number of clinical trials focused on cardiac effects of QCT and its derivatives. Thus, more studies with a stronger design and larger number of enrolled patients for testing different concentrations of QCT are needed to reveal real therapeutic potential of QCT in CVD. Finally, potential doubts based on reported negative effects of QCT should be considered in QCT application; especially proper dosage and application form must represent the golden rule of all QCT-based approaches aimed to treat CVD in humans.

## Figures and Tables

**Table 1:** Overview of QCT derivatives, chemical structures and natural sources.

Chemical Structure	Common Name/ Systematic Name	Food Sources	References
*QCT-3-O-glycosides*			
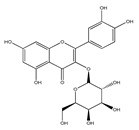	Hyperoside/ QCT-3-O-galactoside	Mango Cranberries Blueberries Chokeberries	[32] [33]
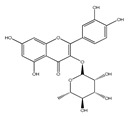	Quercitrin/ QCT-3-O-rhamnoside	Mango Spinach	[32] [34]
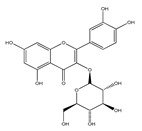	Isoquercitrin/ QCT-3-O- glucoside	Beans Plums Onions Mango	[35] [36] [37] [32]
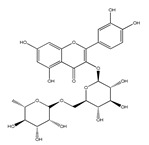	Rutin/ QCT-3-O- rutinoside	Plums Cherries Tomatoes Buckwheat	[36] [38] [39] [40]
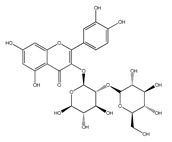	QCT-3-O-sophoroside	Broccoli	[41]
*QCT-7-O-glycosides*			
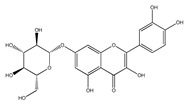	QCT- 7-O-glucoside	Beans	[35]
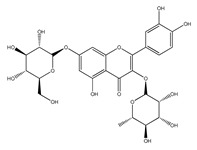	QCT- 3-O-rhamnoside- 7-O-glucoside	Pepper	[42]
*Acyl and sulfate QCT glycosides*			
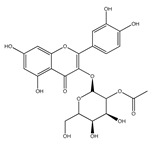	QCT- 3-O-(2″-acetylgalactoside)	*Hypericum perforatum*	[43]
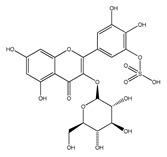	QCT-3-O-glucoside-5′-sulfate	Cornflower	[44]
*QCT-C-glycosides*			
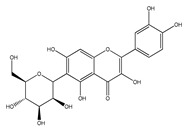	QCT-6-C-glucoside	*Ageratina calophylla*	[25]
*QCT ethers*			
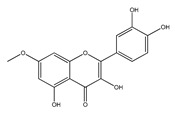	Rhamnetin	*Rhamnus petiolaris*	[45]
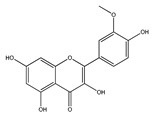	Isorhamnetin	Onions Honey	[46] [47]
*QCT prenyls*			
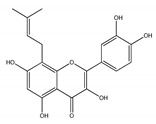	8-prenyl-QCT	*Desmodium caudatum*	[48]

**Table 2:** Summary of potential cardioprotective effects of QCT and its derivatives.

Derivative	Dose	Exp. Model	Type of Injury	Effect	Mechanism	Reference
**Quercetin** **(QCT)**	1–250 mg/kg	Rodents (mice/rats)	I/R	↓oxidative stress ↓inflammation ↓infarct size ↑heart function	↓ROS, ↓HMGB1, ↓NF-kB, ↓TNF- α, ↓apoptosis ↑PI3K/Akt, ↑SIRT1/PGC-1α	[14,16,78,91,95,96,97,102]
	20 mg/kg	Rats	Isoproterenol- induced MI	↓oxidative stress ↓inflammation	↓ROS, ↓calpain	[86]
	0.2% in food	Mdx/Utrn^+/−^ mice	Duchenne muscular dystrophy	↑mitochondrial function	↓NF-kB, ↓TGF-β1, ↓F4/80	[87]
	10 mg/kg	Rats	Autoimmune myocarditis	↓oxidative stress	↓ROS, ↓ER stress, ↑endothelin-1/MAPK	[88]
	10–50 mg/kg	Rats	Diabetic cardiomyopathy	↓oxidative stress ↓cardiac injury ↓inflammation ↓apoptosis	↓troponin C, ↓CK-MB, ↓LDH, ↓ROS ↓ Bax, ↓caspases-3,-9	[103]
	10–80 µM	Cell cultures (H9c2, NRCM)	I/R	↑cell viability ↓oxidative stress ↑mitochondrial function	↓ROS, ↓JNK, ↓p38, ↓MAPK, ↑Bcl-2/Bax, ↑PKCε	[73,74]
	0.1–10 µM	H9c2	4-hydroxynonenal – induced toxicity	↓oxidative stress ↑cell viability	↓ROS, ↓p-SAPK/JNK, ↓p-HSP27, ↓caspase 3	[69]
	500–200 µM	H9c2	Doxorubicin – induced toxicity	↑cell viability ↓ inflammation	↓Src kinase activity, ↓ROS, ↓STAT3	[70]
	100µM	H9c2	H_2_O_2_ – induced toxicity	↓oxidative stress ↑cell viability	↓ROS, ↓P38, ↓MAPK, ↓JNK	[84]
**Troxerutin**	150 mg/kg	Rats	I/R	↓infarct size ↑cardiac function ↓arrhythmias ↓inflammation	↑PI3K/Akt, ↓TNF-α, ↓IL-1b, ↓ICAM-1	[90,100,104]
**Hyperoside**	0.5–50 µM	NRCMs	I/R	↑cell viability	↓Bnip3	[82]
**IsoQCT**	20–80 µM/ml	H9c2	I/R	↑cell viability ↓cell apoptosis mitochondrial protection	↓ROS generation ↓cytochrome c release	[83]
	80 mg/kg	Rats	AMI	↓inflammation ↓apoptosis	↓TLR4-NF-kB	[98]
**Isorhamnetin**	10–40µM	NRCMs	I/R	↓oxidative stress mitochondrial protection	↓ mPTP opening, ↓caspase-3 activity, ↓cytochrome c release, ↓ROS	[79]
**DihydroQCT**	2,5–80 µM	H9c2	I/R	↓oxidative stress ↓apoptosis	↓ROS, ↓ER stress, ↑PI3K/Akt	[80]
	5–20 µM in K-H	Rats	I/R	↓oxidative stress ↓ apoptosis	↓ROS, ↓ER stress, ↑PI3K/Akt	[80]
**ZYZ-772**	1–50 µM	H9c2	CoCl_2_ – induced H/R	↑cell viability ↓oxidative stress ↓ apoptosis	↓ROS, ↓Nox4/MAPK/p53	[81]

Abbreviations: I/R—ischemia/reperfusion; H/R—hypoxia/reoxygenation, MI—myocardial infarction; AMI—acute myocardial infarction; NRCM—neonatal rat cardiac myocytes; K-H—Krebs-Henseleit buffer, ROS—reactive oxygen species, ER – endoplasmic reticulum, LDH—lactate dehydrogenase, JNK—c-Jun-N-terminal kinase, PI3K—phosphoinositide 3-kinase, Akt—protein kinase B, Bcl-2 – B-cell lymphoma 2; Bax—Bcl-2-associated X protein, MAPK—mitogen-activated protein kinase, ICAM-1—intercellular adhesion molecule 1, TLR4—toll-like receptor 4, mPTP—mitochondrial permeability transition pore, TNF-α—tumor necrosis factor α, Bnip3—Bcl-2 nineteen-kD interacting protein 3, Nox4—NADPH oxidase 4, SIRT1—Silent information regulator 1, PGC-1α—peroxisome proliferator initiated receptor gamma and coactivator 1 alpha.
